# Technical Tips and Efficacy of Leadless Pacemaker Implantation in a Patient With Isolated Persistent Left Superior Vena Cava

**DOI:** 10.1002/joa3.70149

**Published:** 2025-07-18

**Authors:** Yuki Tokonami, Makoto Sano, Taro Narumi, Yoshihisa Naruse, Yuichiro Maekawa

**Affiliations:** ^1^ Division of Cardiology, Internal Medicine III Hamamatsu University School of Medicine Hamamatsu Japan

**Keywords:** atrioventricular synchronous pacing, isolated persistent left superior vena cava, leadless pacemaker

## Abstract

Isolated persistent left superior vena cava (PLSVC) provides limited access during transvenous pacemaker implantation. We present a case of implantation of a leadless pacemaker for atrioventricular block with isolated PLSVC. A leadless pacemaker is an alternative strategy to a transvenous pacemaker; however, some technical tips are required: (1) the prevention of the mis‐insertion in the dilated coronary sinus and (2) the pre‐assessment of the anatomy of the right atrium and ventricle.

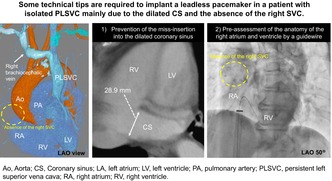

An 80‐year‐old female with dyspnea on exertion was admitted to a local hospital. Her medical history included type 2 diabetes mellitus and atopic dermatitis. The patient's height, weight, and body mass index were 155.0 cm, 43.5 kg, and 18.1 kg/m^2^, respectively. She was prescribed oral hypoglycemic agents. Her blood pressure was 175/47 mmHg, heart rate was 44 beats/min, and oxygen saturation was 97% on room air. Physical and laboratory examinations revealed lower limb edema, blood urea nitrogen of 20.2 mg/dL, creatinine of 0.66 mg/dL, hemoglobin of 10.6 g/dL, NT‐proBNP of 655 pg/mL, and troponin T of 0.01 ng/mL. Chest radiography revealed bilateral lung congestion. Twelve‐lead electrocardiography (ECG) revealed a heart rate of 44 beats/min and a 2:1 atrioventricular block without ST‐T changes. Echocardiography revealed normal contraction of both ventricles with a left ventricular ejection fraction of 67.6%. Preprocedural venograms of the left and right upper extremities revealed a persistent left superior vena cava (PLSVC) without the right superior vena cava. This congenital anomaly is termed an isolated PLSVC. The patient was transferred to our hospital for the implantation of a leadless pacemaker (Micra AV). A preoperative computed tomography (CT) scan provided information on the isolated PLSVC and right atrium (RA) anatomy (Figure 1A–C).

**FIGURE 1:**
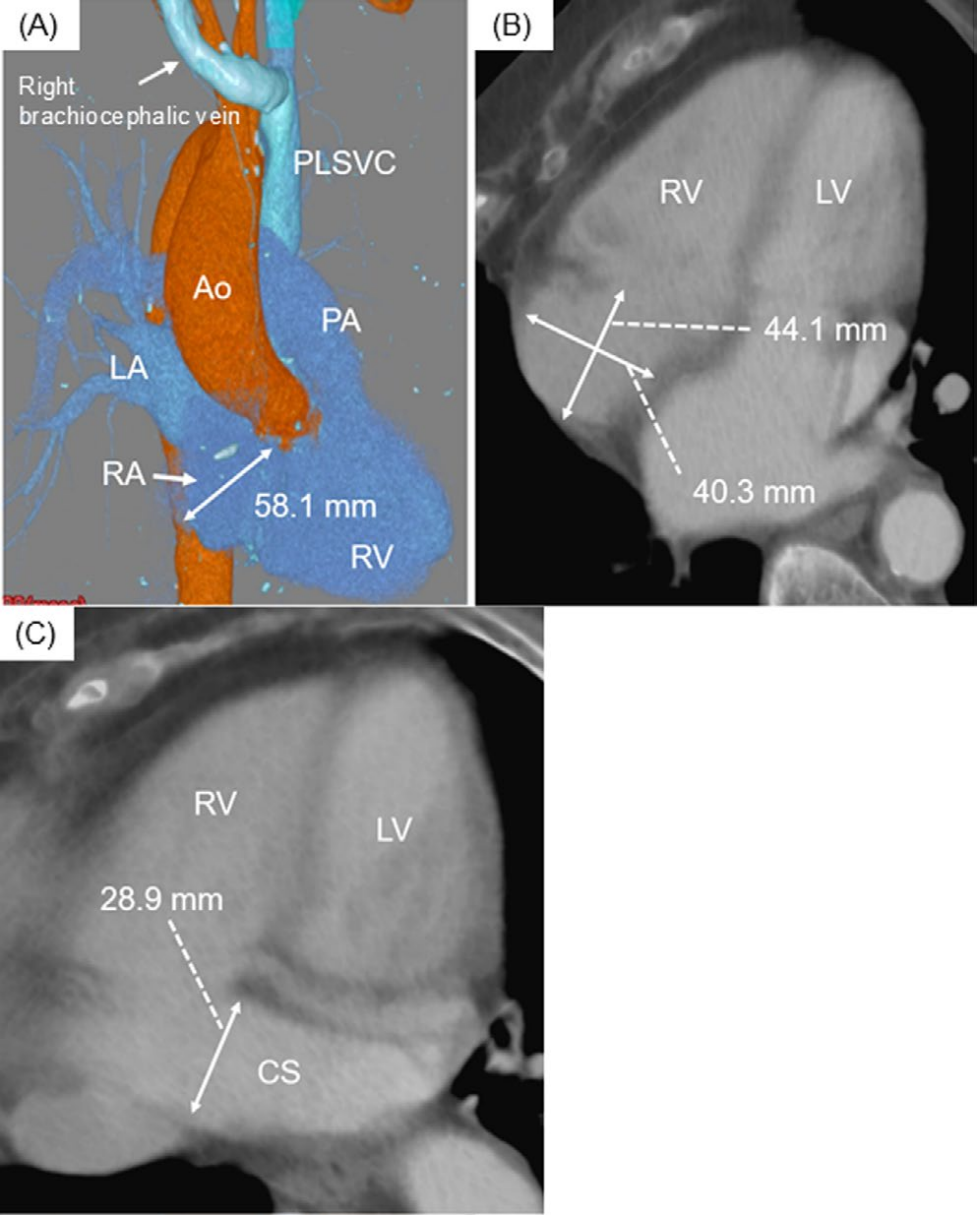
(A) A volume rendering CT image in the RAO view revealed isolated PLSVC and the distance from the IVC outlet to the superior edge of the tricuspid valve annulus is 58.1 mm. (B) The long and transverse diameters of the RA in the horizontal four‐chamber view were 44.1 and 40.3 mm, respectively. (C) The diameter of the dilated coronary sinus was 28.9 mm. Ao, aorta; CS, coronary sinus; CT, computed tomography; IVC, inferior vena cava; LA, left atrium; LV, left ventricle; PA, pulmonary artery; PLSVC, persistent left superior vena cava; RA, right atrium; RAO, right anterior oblique; RV, right ventricle.

Vascular access was obtained via the right femoral vein under mild sedation using midazolam and local anesthesia. Right atriography demonstrated the RA size and the direction from the RA to the right ventricle (RV) (Figure 2A,B and Videos S1 and S2). The guidewire was turned back at the roof of the RA and inserted into the RV (Figure 2C). The Micra introducer was carefully inserted into the RA and the delivery catheter was gently pushed to the right ventricular septal wall (Figure 2D). In a second attempt, Micra AV was successfully implanted with stable electrical performance. The patient was discharged 7 days after implantation. Device interrogation 6 months after implantation showed 77.3% atrioventricular synchrony.

**FIGURE 2:**
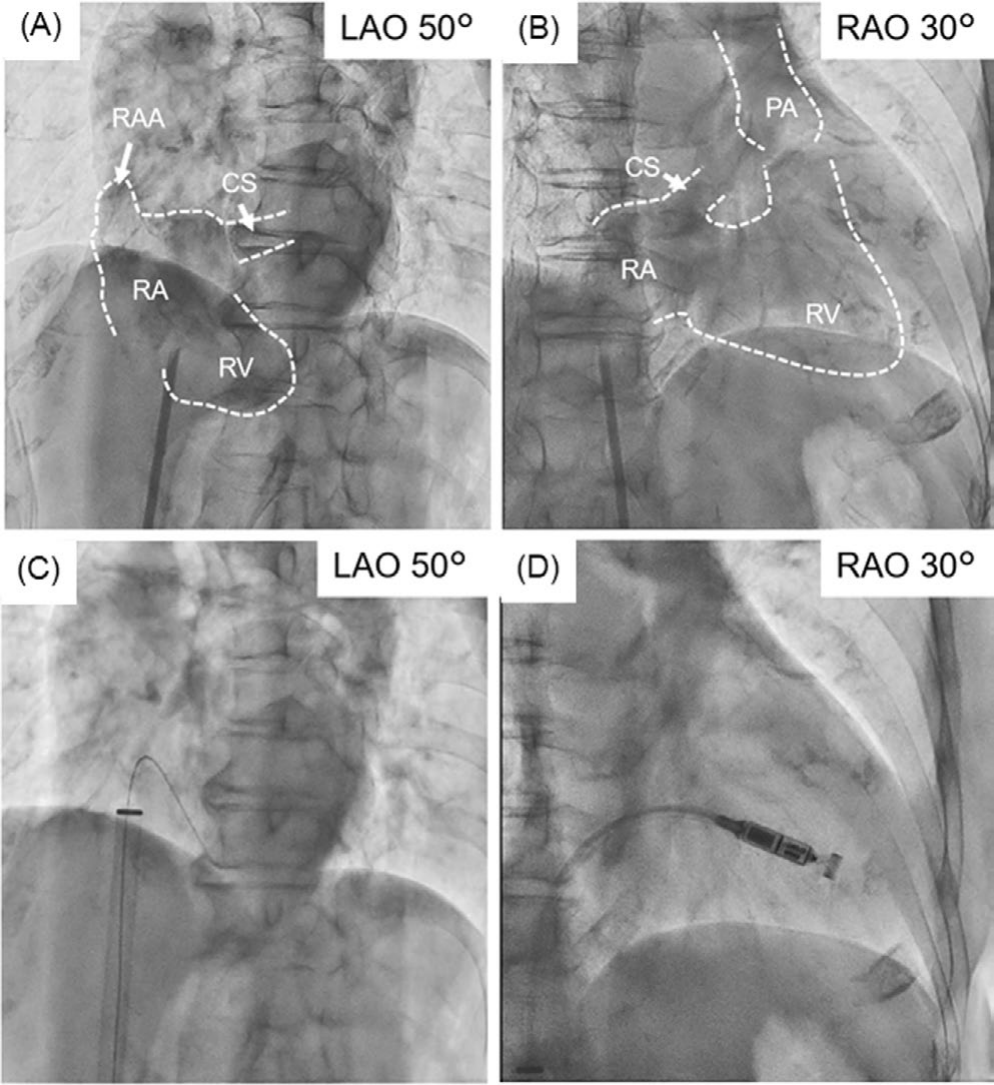
The fluoroscopic view during Micra implantation. (A) and (B) showed the RAG images in the LAO and RAO views, respectively, revealing that the right SVC did not exist. (C) The guidewire was inserted into the RV in the LAO fluoroscopic view. (D) The delivery catheter was mildly pushed into a gooseneck shape in the RAO view. CS, coronary sinus; LAO, left anterior oblique; PA, pulmonary artery; RA, right atrium; RAA, right atrial appendage; RAG, right atriography; RAO, right anterior oblique; RV, right ventricle; SVC, superior vena cava.

Isolated PLSVC occurs in 0.07%–0.13% of cases of congenital heart disease [1]. This anomaly is characterized by a PLSVC draining into the coronary sinus (CS) and an azygos vein draining into the PLSVC [1]. Isolated PLSVC is typically asymptomatic. It is problematic during procedures such as transvenous pacemaker implantation, central venous catheter insertion, and cardiac surgery [1].

Some case reports have described the successful transvenous pacemaker implantation in patients with isolated PLSVC via the left subclavian vein, PLSVC, CS, or RA [2]. A unique technique involving a shaping stylet was required to approach the RA and the RV through a tortuous venous course. However, the long‐term durability of transvenous leads and their adverse effects on the CS remain unclear. In contrast, leadless pacemaker implantation is an alternative strategy in cases where the access vein has stenosis or occlusion, such as isolated PLSVC.

Preoperative imaging modalities are useful for understanding the anatomy of the RA. Roberto et al. reported that the normal references of indexed long and transverse diameter on a horizontal four‐chamber view with magnetic resonance imaging were 3.2 ± 0.4 and 2.7 ± 0.3 cm/m^2^ (mean ± SD), respectively [3]. They also reported that the normal diameter of the CS ostium was 9–15 mm. Soejima et al. showed that the distance from the IVC outlet to the superior edge of the tricuspid valve annulus was 58.4 ± 9.0 mm in Japanese patients [4]. These parameters were 44.1 mm (3.20 cm/m^2^), 40.3 mm (2.92 cm/m^2^), 28.9 mm, and 58.1 mm in our patient, as measured on CT images (Figure 1B,C). Therefore, the RA size in our patient may have been normal; however, the CS was dilated extensively. Right atriography was also important to confirm the precise anatomy of the RA roof and CS during the Micra implantation procedure (Figure 2A,B, and Videos S1 and S2). Specific procedural techniques are required to implant a leadless pacemaker via the femoral vein in patients with an isolated PLSVC. Remarkable abnormalities were the absence of the right SVC and a dilated CS. Particular attention was paid to the following three procedures: (1) insertion of a guidewire into the RV (not the SVC), (2) insertion of the Micra introducer into the RA, and (3) insertion of the Micra introducer into the RV. First, the guidewire was inserted into the RV, turning back at the roof of the RA, because it could not be inserted into the right SVC. This procedure was also useful for confirming the RA roof and the direction from the RA to the RV. The guidewire tended to be inserted into the CS because of the dilated CS. Second, the Micra introducer could not be inserted at the mid‐level of the RA because the inner (dilator) protruded 140 mm from the outer (introducer). The outer was then gently pushed into the RA through the inner. Lastly, the dilated CS ostium could cause inserting the Micra introducer into the CS accidentally. To avoid it, confirming the correct direction from RA to RV was required. Right atriography could visualize the anatomy of RA, the CS, and the RV. The guidewire inserted into RV also provide an anatomical information. The other maneuvers, such as pushing the delivery catheter, releasing the Micra, recapturing the Micra, and retrieving the delivery catheter, could be performed in the same manner as in normal anatomy.

Micra AV provide synchronous atrioventricular pacing via mechanical atrial sensing. The rate of atrioventricular synchrony is 70%–90% in normal anatomy [5]. It is unclear whether an abnormal RA anatomy affects mechanical atrial sensing. In the present case, atrioventricular synchrony was achieved despite abnormal RA anatomy. It is suspected that blood flow from the RA to the RV is the same as that under normal anatomical conditions because the tricuspid valve, annulus, and RV are normal structures, even with isolated PLSVC. Lead implantation via the left subclavian vein, PLSVC, and CS may pose a potential risk of severe lead‐related adhesions in the vein, making lead extraction difficult when considering lead management. However, coronary malperfusion is not expected to occur because of a dilated CS.

Implantation of a transvenous dual‐chamber pacemaker in a patient with isolated PLSVC is challenging because of the tortuous venous course, and the transvenous lead may make lead extraction difficult. By contrast, leadless pacemakers can be successfully deployed through the femoral vein. Specific procedural techniques are required, such as focusing on the absence of the right SVC and the dilated CS. Micra AV also achieved acceptable atrioventricular synchronous pacing in our patient with isolated PLSVC.

## Ethics Statement

The authors have nothing to report.

## Consent

The authors confirm that written consent for the submission and publication of this case report was obtained from the patient in line with COPE guidance.

## Conflicts of Interest

The authors declare no conflicts of interest.

## Supporting information


**Video S1.** The right atriography in the LAO view demonstrated the RA size and the direction from the RA to the right ventricle. LAO, left anterior oblique; RA, right atrium.

**Video S2.** The right atriography in the RAO view demonstrated the RA size and the direction from the RA to the right ventricle. RA, right atrium; RAO, right anterior oblique.

## Data Availability

Raw data were generated at the Hamamatsu University School of Medicine. The data supporting the findings of this study are available from the corresponding author upon request.
